# Supplementary material for: Splice-Junction-Based Mapping of Alternative Isoforms in the Human Proteome
Source: Cell Rep. Author manuscript; Available in PMC 2020 Jan 15. (PMC6961840; doi:10.1016/j.celrep.2019.11.026)
Supplement: 3 [file NIHMS1546469-supplement-3.zip › DF2/PXD0010154/Lung-296-Q16827-DGKLPYNWSK.pdf]

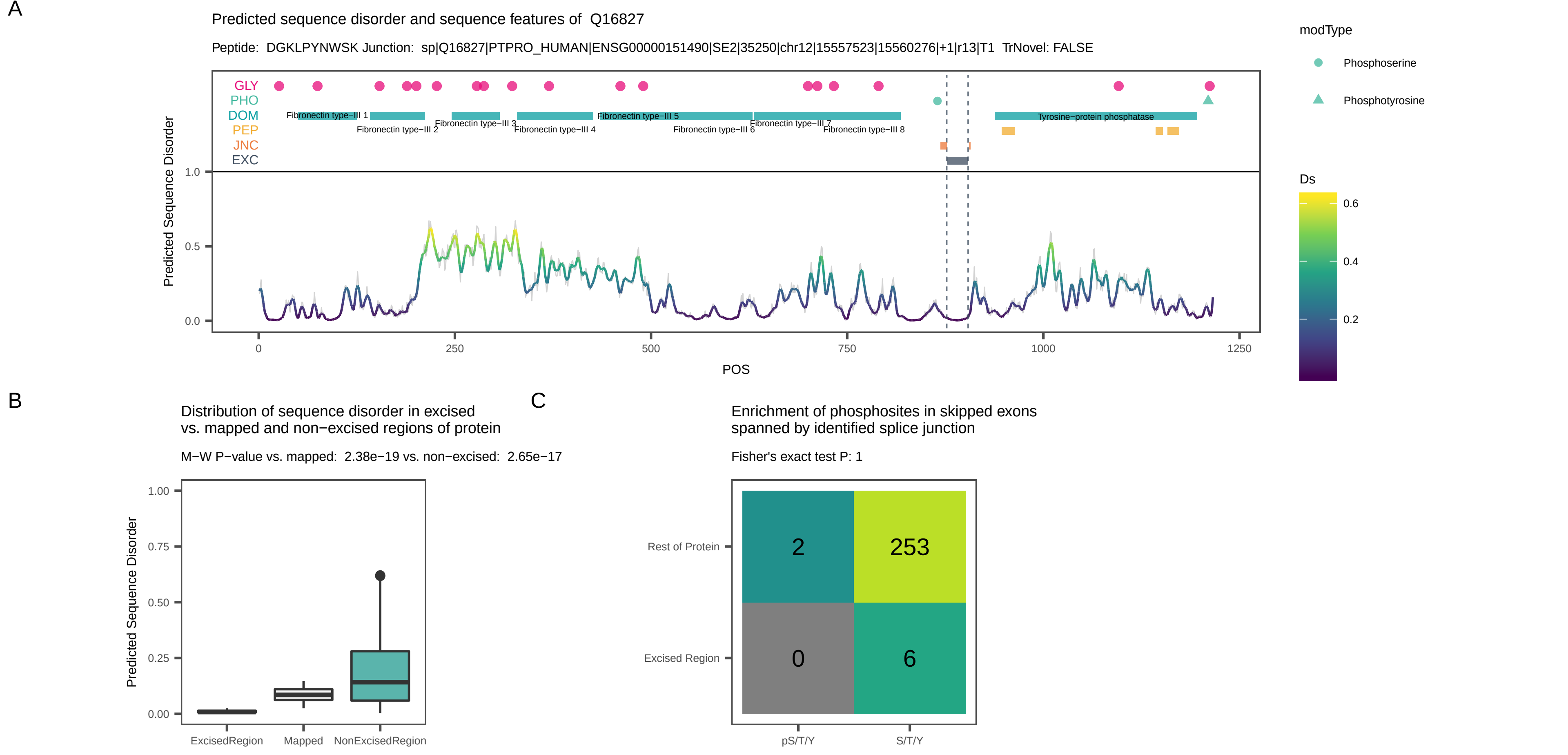

A
Predicted sequence disorder and sequence features of Q16827
Peptide: DGKLPYNWSK Junction: sp|Q16827|PTPRO_HUMAN|ENSG00000151490|SE2|35250|chr12|15557523|15560276|+1|r13|T1 TrNovel: FALSE
GLY
PHO
DOM
PEP
JNC
EXC
Fibronectin type−III 1
Fibronectin type−III 2
Fibronectin type−III 3
Fibronectin type−III 4
Fibronectin type−III 5
Fibronectin type−III 6
Fibronectin type−III 7
Fibronectin type−III 8
Tyrosine−protein phosphatase
Predicted Sequence Disorder
1.0
0.5
0.0
0
250
500
750
1000
1250
POS
modType
Phosphoserine
Phosphotyrosine
Ds
0.6
0.4
0.2
B
Distribution of sequence disorder in excised
vs. mapped and non−excised regions of protein
M−W P−value vs. mapped: 2.38e−19 vs. non−excised: 2.65e−17
Predicted Sequence Disorder
1.00
0.75
0.50
0.25
0.00
ExcisedRegion
Mapped
NonExcisedRegion
C
Enrichment of phosphosites in skipped exons
spanned by identified splice junction
Fisher's exact test P: 1
Rest of Protein
Excised Region
2
253
0
6
pS/T/Y
S/T/Y
